# Niclosamide suppresses acute myeloid leukemia cell proliferation through inhibition of CREB-dependent signaling pathways

**DOI:** 10.18632/oncotarget.23794

**Published:** 2017-12-31

**Authors:** Hee-Don Chae, Nick Cox, Gary V. Dahl, Norman J. Lacayo, Kara L. Davis, Samanta Capolicchio, Mark Smith, Kathleen M. Sakamoto

**Affiliations:** ^1^ Division of Hematology/Oncology, Department of Pediatrics, Stanford University School of Medicine, Stanford, CA, USA; ^2^ Medicinal Chemistry Knowledge Center, Stanford ChEM-H, Stanford, CA, USA

**Keywords:** niclosamide, acute myeloid leukemia, small molecule, CREB, combination

## Abstract

CREB (cAMP Response Element Binding protein) is a transcription factor that is overexpressed in primary acute myeloid leukemia (AML) cells and associated with a decreased event-free survival and increased risk of relapse. We recently reported a small molecule inhibitor of CREB, XX-650-23, which inhibits CREB activity in AML cells. Structure-activity relationship analysis for chemical compounds with structures similar to XX-650-23 led to the identification of the anthelminthic drug niclosamide as a potent anti-leukemic agent that suppresses cell viability of AML cell lines and primary AML cells without a significant decrease in colony forming activity of normal bone marrow cells. Niclosamide significantly inhibited CREB function and CREB-mediated gene expression in cells, leading to apoptosis and G1/S cell cycle arrest with reduced phosphorylated CREB levels. CREB knockdown protected cells from niclosamide treatment-mediated cytotoxic effects. Furthermore, treatment with a combination of niclosamide and CREB inhibitor XX-650-23 showed an additive anti-proliferative effect, consistent with the hypothesis that niclosamide and XX-650-23 regulate the same targets or pathways to inhibit proliferation and survival of AML cells. Niclosamide significantly inhibited the progression of disease in AML patient-derived xenograft (PDX) mice, and prolonged survival of PDX mice. Niclosamide also showed synergistic effects with chemotherapy drugs to inhibit AML cell proliferation. While chemotherapy antagonized the cytotoxic potential of niclosamide, pretreatment with niclosamide sensitized cells to chemotherapeutic drugs, cytarabine, daunorubicin, and vincristine. Therefore, our results demonstrate niclosamide as a potential drug to treat AML by inducing apoptosis and cell cycle arrest through inhibition of CREB-dependent pathways in AML cells.

## INTRODUCTION

Acute myeloid leukemia (AML) is an aggressive hematologic malignancy that is characterized by the clonal accumulation of immature myeloid progenitors, leading to multilineage cytopenia with a poor outcome [1–4]. As AML results from the accumulation of several oncogenic hits [5], it is a complex disease with genetic and clinical heterogeneity [3]. The cAMP response element binding protein (CREB), a stimulus-induced transcription factor that responds rapidly to phosphorylation and co-activator activation, has been implicated in a number of cancers, including leukemia [6–8]. Upon phosphorylation at Ser133 in its Kinase Inducible Domain (KID) domain, CREB recruits co-activator CREB binding protein (CBP) through interaction between the KID interacting (KIX) domain and the CREB KID domain [9] to induce expression of CREB-driven genes that regulate cell proliferation, signal transduction, and cell survival [10–12]. Overexpression of CREB contributes to transformation of hematopoietic cells. CREB is overexpressed at a higher frequency in AML patients and associated with a decreased event-free survival and an increased risk of relapse [6]. Furthermore CREB-overexpression in myeloid cells results in a myeloproliferative disorder in CREB-transgenic mice [13]. CREB knockdown inhibits AML cell proliferation, but does not affect normal hematopoietic stem cell activity in mouse transduction and transplantation experiments [14].

The standard of therapy for AML over the last 40 years has been “7+3” combination chemotherapy, with 7 days of cytarabine plus 3 days of an anthracycline, followed by consolidation chemotherapy or hematopoietic cell transplant [2, 3, 15]. Even with intensive chemotherapy, the overall 5-year survival is less than 60% in younger patients and less than 10% in the elderly patients [2, 16]. Therefore, more effective and less toxic approaches to treat AML are needed. We recently demonstrated the feasibility of targeting CREB for AML treatment using XX-650-23, a small molecule inhibitor of CREB function, which is based on naphthol AS-E phosphate that was first identified as an inhibitor of CREB interaction with its coactivator, CREB Binding Protein (CBP) [17]. Although the CREB inhibitor XX-650-23 was more potent than naphthol AS-E phosphate, the small molecule did not have adequate physicochemical properties or sufficient potency for clinical application.

To this end, we performed structure-activity relationship (SAR) studies for chemical compounds with structures similar to XX-650-23. We identified niclosamide, a FDA approved drug with a similar chemical structure that uncouples oxidative phosphorylation in the mitochondria. Niclosamide is an oral anthelminthic drug that has been used worldwide to treat tapeworm infections for 50 years [18–20]. Niclosamide is not toxic in mammals (oral LD50 in rats: over 5000mg/kg) [21] and is well tolerated in humans [22]. Recent studies have proposed that niclosamide has anticancer activity in different cancer types by targeting multiple signaling pathways including NF-kB, Wnt/β-catenin, mTORC1, STAT3, and Notch [20, 23–25].

In this study, we demonstrate that niclosamide inhibits CREB-driven gene expression by preventing CREB activation, thereby disrupting CREB-CBP interaction leading to induction of apoptosis and cell cycle arrest in AML cells. We also investigate the *in vivo* efficacy of niclosamide in AML patient-derived xenograft (PDX) mouse models. Furthermore, combination of niclosamide with chemotherapy showed synergistic effects on AML cells, suggesting that sequential combination of niclosamide with lower doses of “7+3” chemotherapy may provide a more efficacious and less toxic approach to treat AML.

## RESULTS

### Niclosamide suppresses AML proliferation as an inhibitor of CREB-dependent pathway

**Figure 1:**
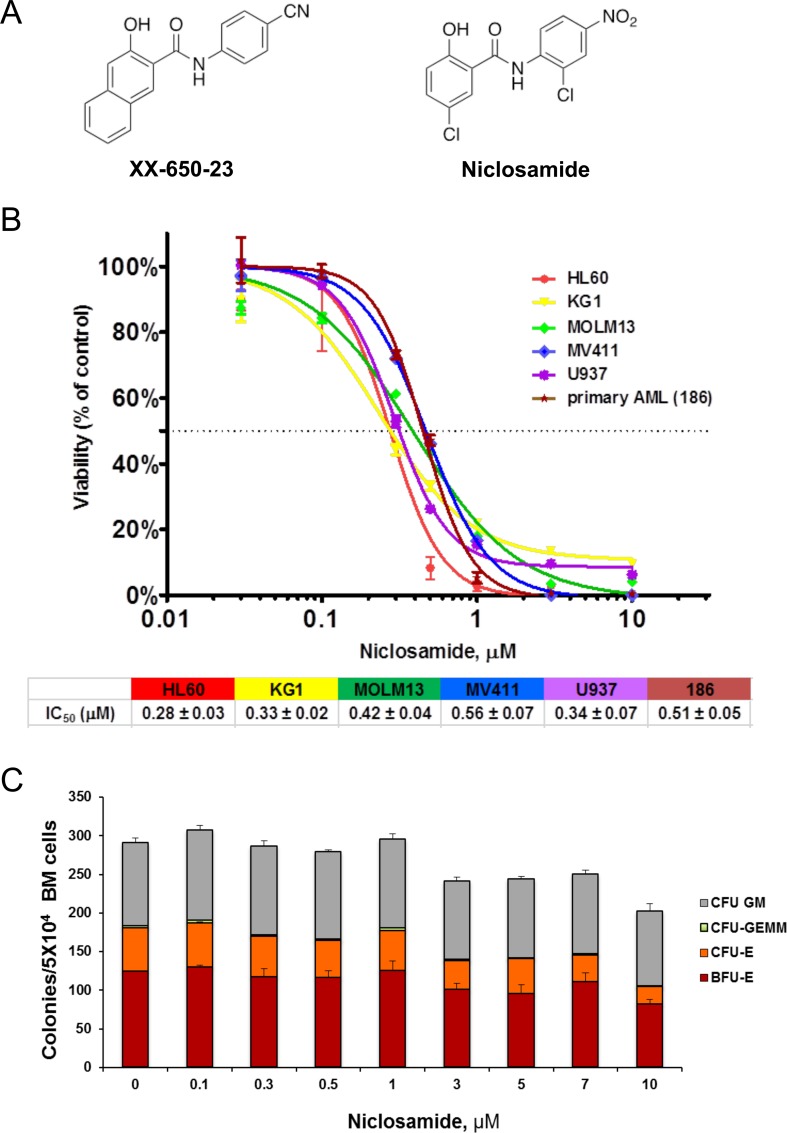
Niclosamide effectively and selectively inhibits viability of AML cells **A.** Chemical structure of XX-650-23 and niclosamide. **B.** AML cells are sensitive to niclosamide. Human AML cell lines and primary AML cells were plated at 2×10^4^ cells/well in 96-well plates, and cultured with niclosamide or vehicle for 3d or 4d, respectively. Cell Titer Glo assay was performed to assess viability of cells. The graphs show representative dose response curves. The IC_50_s are described below the graph (HL60: *n* = 54, KG1: *n* = 34, MOLM13: *n* = 3, MV411: *n* = 3, U937: *n* = 10, 186: *n* = 3). **C.** Effect of niclosamide on normal BM colony forming activity. Normal human bone marrow cells from healthy donors were seeded (3×10^4^ cells/plate) in methylcellulose media with niclosamide, and cultured for 2 weeks. Colonies were scored based on morphology. Data are graphed as mean ± standard error measurement (SEM) (*n* = 3).

XX-650-23 was previously shown to inhibit CREB activity and suppress AML cells proliferation *in vitro* with an IC_50_ of 1-2 μM and a short half-life when injected intraperitoneally in mice. With the goal of identifying a more potent CREB inhibitor with improved pharmacokinetic properties, we synthesized and tested a series of structural analogs to further develop SAR. At the same time, we searched for existing drugs with known pharmacokinetic and safety profiles using 2D chemical similarity analysis methods [26–28]. This effort led to the identification of the FDA-approved anthelmintic niclosamide as a potential inhibitor of CREB-dependent pathways. Niclosamide shares a number of structural features with XX-650-23 (Figure 1A), including the key salicylanilide core, an electron withdrawing group para to the phenol hydroxyl group, and an electron-withdrawing group para to the anilide. Niclosamide has been reported to exert anti-tumor activity in several cancers including AML [20, 23–25]. We examined the effects of niclosamide on cellular viability of AML cell lines and primary human AML cells. Niclosamide significantly inhibited cellular viability in a dose-dependent manner with IC_50_ of 0.28 to 0.51 μM (Figure 1B). We then investigated the cytotoxic effects of niclosamide in normal bone marrow cells. Though the size of colonies became smaller when treated with over 3 μM of niclosamide, colony formation of normal bone marrow cells was not significantly inhibited up to 10 M of niclosamide (18- to 36-fold therapeutic window, Figure 1C). Colony-forming unit-erythroid (CFU-E) was shown to be more vulnerable to niclosamide due to its smaller colony size.

**Figure 2:**
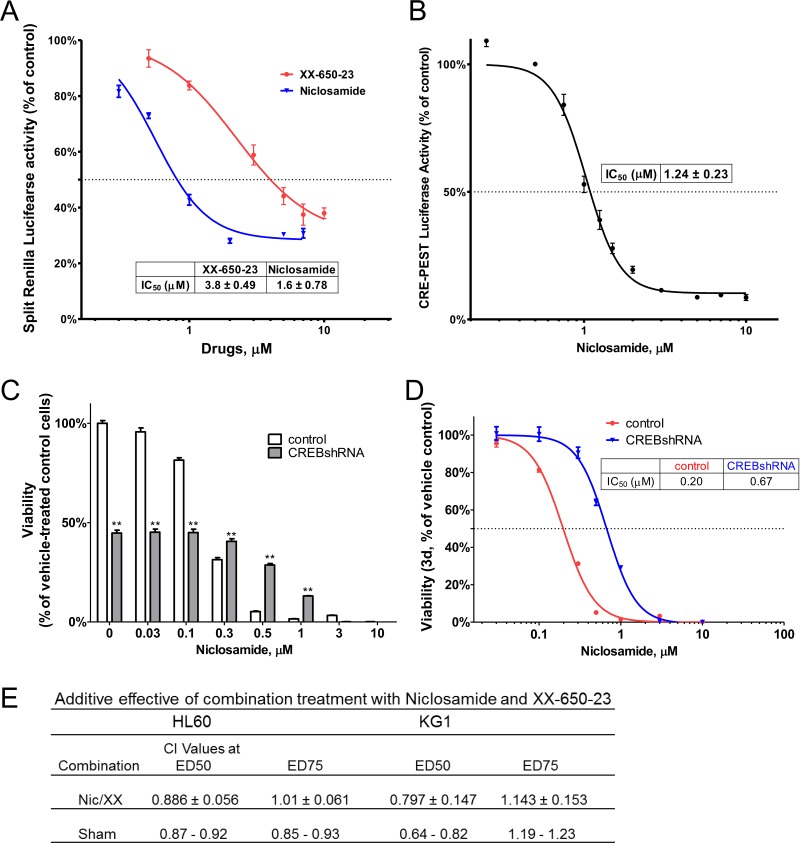
Niclosamide inhibits CREB-dependent pathways **A.** Niclosamide was more potent than XX-650-23 to inhibit CBP KIX-CREB KID domain interaction. RLucC-KIX and KID-RLucN expressing vectors were transfected into 293 cells. Transfected 293 cells were treated with compounds 30 minutes before forskoin (6 μM) stimulation. Cells were further incubated for 90 minutes, and measured Renilla luciferase activity using coelenterazine afterwards. Data are presented as mean ± SEM (*n* = 3). **B.** HL60 cells expressing CREB-driven luciferase were generated. Cells were treated with niclosamide for 6h. Luciferase activity was significantly inhibited by niclosamide treatment. Data are presented as mean ± SEM (*n* = 3). The IC_50_s are described in the graphs. **C.** CREB knockdown AML cells were more resistant to niclosamide. HL60 cells were transduced with CREB or control shRNA lentivirus. Transduced cells were treated with niclosamide for 3 days. Cell Titer Glo assays were performed. The graph shows that CREB knockdown itself inhibited cellular viability of HL60 cells. Values are indicated as mean ± SEM (*n* = 3). **, *p* < .01. **D.** Viability dose-response curve for CREB shRNA transduced cells shifted to the right, suggesting more resistant cells. **E.** Additive effect of combination treatment with niclosamide and XX-650-23 in HL60 and KG1 cells. Cells were treated with various concentrations of niclosamide (Nic) and XX-650-23 (XX) for 3d. Viability was accessed using CellTiter-Glo assay kit. Combination index (CI) values were calculated by Chou-Talalay method using CalcuSyn software. Data are the mean ± SEM (*n* = 4). Additivity ranges of CI scores were determined from sham mixtures of the same compound (niclosamide or XX-650-23) in each cell line.

Next, we evaluated whether niclosamide could inhibit CREB activation and subsequent interaction of CREB with CBP. CREB-CBP interaction is dependent on CREB phosphorylation at serine 133. Using the Renilla luciferase complementation assay, we determined niclosamide to be more potent than XX-650-23 as an inhibitor of the CBP KIX-CREB KID domain interaction in a dose dependent manner (IC_50_: 1.6 μM *vs*. 3.8 μM) (Figure 2A). We next investigated whether CREB inhibition could block CREB-dependent transcription using a CREB-driven luciferase reporter assay in HL60 cells. We used a reporter gene construct expressing luciferase under the control of a promoter containing two cyclic AMP response elements (CRE). Luciferase activity was measured after treatment of HL60 cells with niclosamide for 6 hours. Consistent with the inhibition of KIX-KID interaction, niclosamide reduced CREB-driven luciferase activity in a dose-dependent manner with an IC_50_ of 1.24 ± 0.23 μM (Figure 2B). To examine the functional requirement of CREB, we determined the effects of CREB knockdown in HL60 cells treated with niclosamide. Knockdown of the cytotoxic drug target could mimic cellular inhibitory phenotype of the drug, and conferred resistance to cytotoxic drug activity [29, 30]. While CREB knockdown itself suppressed the viability of cells to approximately 50%, niclosamide, up to 0.3 μM, did not further decrease viability of CREB knockdown cells suggesting that CREB is required for maximal activity of niclosamide in HL60 cells (Figure 2C). CREB knockdown significantly shifted the dose response curve to the right (IC_50_=670 nM for CREB knockdown compared to 200nM for vector control cells), suggesting that CREB is among the targets in cytotoxic response to niclosamide (Figure 2D).

Drug combinations regulating the same target or different targets in the same pathway have been reported to result in additive effects [31]. Thus, we assessed the effects of niclosamide and the CREB inhibitor XX-650-23. If additive, the range of combination index (CI) scores should be determined from sham mixtures of the same compound (niclosamide or XX-650-23) [32]. Though CI values were less than 1 at ED_50_, they were still in the range of sham mixtures, demonstrating that combination of niclosamide and XX-650-23 was additive in AML cells (Figure 2E). These data suggest that niclosamide is exerting its anti-leukemic effects by inhibiting CREB function similar to XX-650-23.

### Niclosamide induces apoptosis and G1/S arrest with suppressed CREB activation

**Figure 3:**
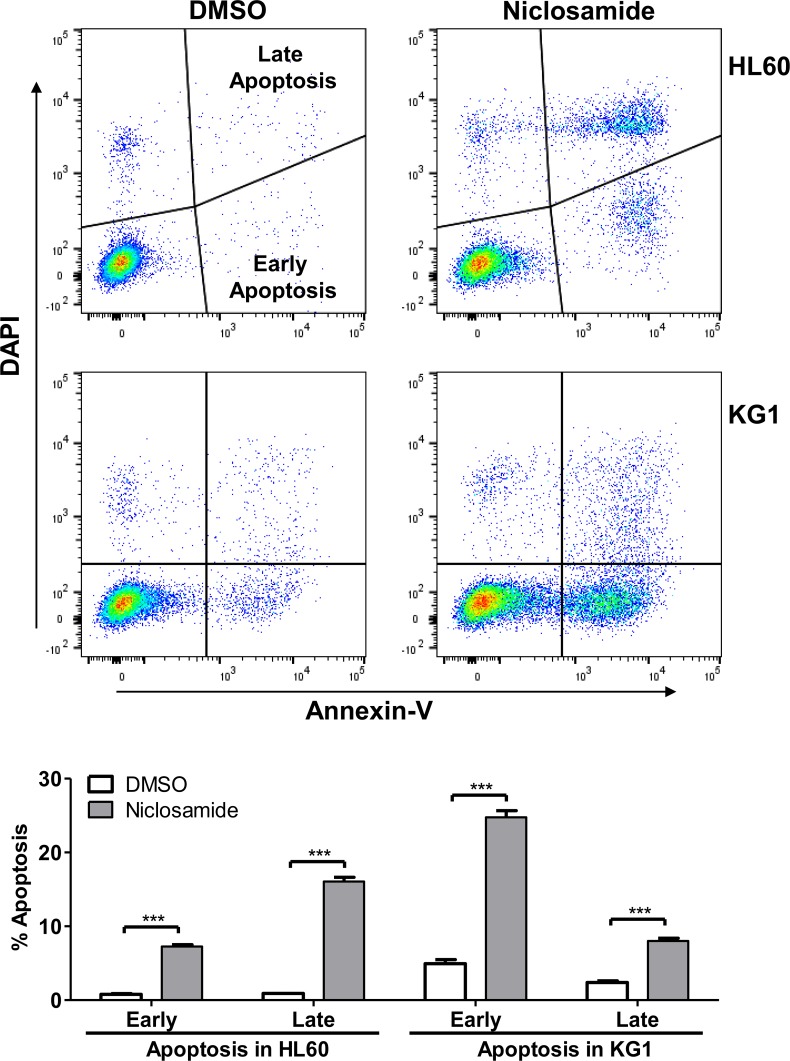
Niclosamide induces apoptosis in AML cells HL60 (top panels) and KG1 (bottom panels) cells were treated with niclosamide (1 μM, 24 h). Apoptotic cells were assessed by Annexin-V/DAPI staining. Early apoptotic (Annexin-V^+^/DAPI^−^) and late apoptotic (Annexin-V^+^/DAPI^+^) populations were significantly increased following treatment with niclosamide. Plots are representative of three independent experiments. Percentages of early and late apoptotic cells are graphed as mean ± SEM (*n* = 3). ***, *p* < .0001.

**Figure 4:**
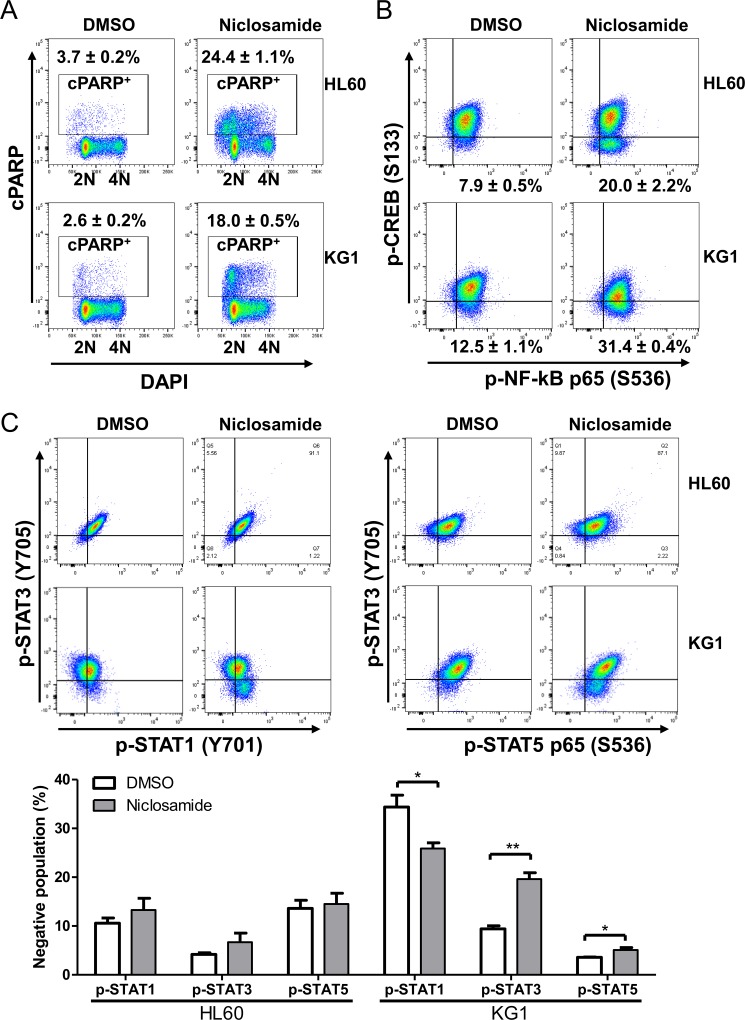
Niclosamide treatment induces apoptosis and suppresses CREB activation HL60 and KG1cells were treated with 1 μM niclosamide for 1d. **A.** Cells were fixed, and then stained with anti-cleaved PARP (cPARP) antibody and DAPI. Percentages of apoptotic cells are indicated. **B.** Fixed cells were stained with anti-phospho-CREB(p-CREB) (S133) and anti-phospho-NFkB p65 (S536) antibodies. Plots show the suppression of p-CREB in cells treated with niclosamide as compared with untreated control, but not phospho-NFkB p65. Percentages of p-CREB-negative population are presented as mean ± SEM (*n* = 3). **C.** Cells were stained with anti-phospho-STAT1 (Y701), anti-phospho-STAT3 (Y705), and anti-phospho-STAT5 (Y694) antibodies. HL60 cells: Top panels, KG1 cells: Bottom panels. Negative populations of each antibody were calculated. Values are graphed as mean ± SEM (*n* = 3). *, *p* < .05; **, *p* < .001.

To identify the mechanism of niclosamide-induced cytotoxicity, we performed apoptosis and cell cycle analysis of niclosamide-treated cells by Annexin-V/DAPI double staining. Induction of apoptosis at early (Annexin-V^+^/DAPI^-^) and late (Annexin-V^+^/DAPI^+^) stages was clearly observed after 1day treatment of niclosamide in HL60 and KG1 cells (Figure 3). While most apoptotic cells were at early stage in KG1 cells, HL60 cells showed higher portion of late apoptosis. Since PARP (poly(ADP-ribose) polymerase) is cleaved by caspase-3 during apoptosis [33], cleaved PARP (cPARP) level was determined by flow cytometry to assess caspase-dependent apoptosis. Percentage of cPARP-positive cells was distinctly increased 1d after niclosamide treatment in both cells (Figure 4A). NF-κB signaling has been reported as a target molecule of niclosamide in several cancer cells [23, 25]. To determine the role of CREB and NF-κB in response to niclosamide, we assessed levels of activated phosphorylated CREB [p-CREB(S133)]and NF-κB p65 [p-NF-κba;B p65 (S536)] in HL60 and KG1 cells by flow cytometry. Niclosamide treatment significantly increased percentage of p-CREB (133)-negative cells, but not p-NF-κB p65 (S539)-negative cells (Figure 4B).

As niclosamide has been known to be a STAT3 inhibitor [23, 34], we measured the phosphorylated protein levels of STAT1 (Y701), STAT3 (Y705), and STAT5 (Y694) by flow cytometry. While the percentage of tyrosine-phosphorylated STAT3 (Y705) -negative population was decreased by 8.5% in KG1 cells, tyrosine-phosphorylated STAT1 (701) and STAT5 (Y694) expressed cell populations were increased following niclosamide treatment in KG1 cells. However, we did not observe any differences in the levels of phosphorylated STAT1 (pY705), STAT3 (pY694), and STAT5 (pY701) protein by niclosamide treatment in HL60 cells (Figure 4C). These results suggest that inhibition of STAT3 is not a direct mechanism of niclosamide effect.

**Figure 5:**
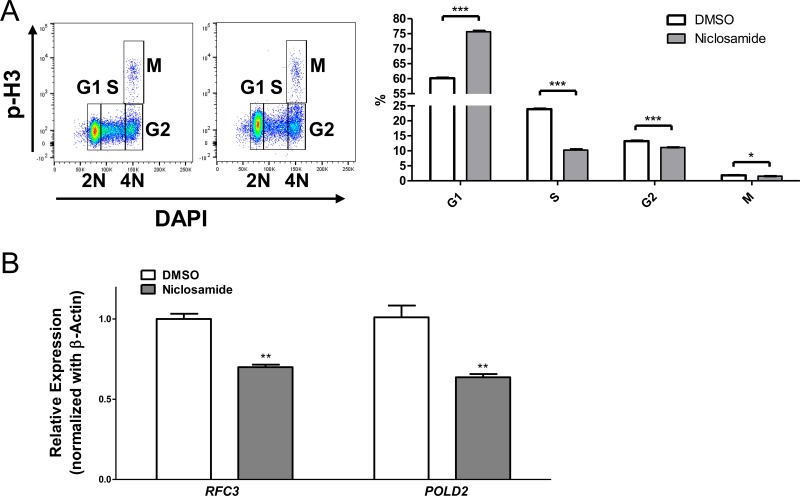
Niclosamide treatment induces G1/S arrest **A.** HL60 cells were treated with niclosamide (1 μM) for 1d. Cells were fixed, and then stained with anti-cPARP, anti-Cyclin A, anti-phospho-Histone H3 (p-H3) antibodies, and DAPI. c-PARP-negative live cells population was gated. p-H3 *versus* DNA content displayed a typical cell cycle profile. Percentage of cells at each cell cycle phase was calculated. Values are indicated as mean ± SEM (*n* = 3). *, *p* < .05; ***, *p* < .001. **B.** Downregulation of CREB-target genes *RFC3* and *POLD2* following treatment of niclosamide (1 μM) for 1d. *RFC3* and *POLD2* mRNAs were assessed by RT-qPCR and normalized against β-actin expression level. The data represent means of three independent experiments ± SEM. **, *p* < .01.

We next examined the effects of niclosamide on the cell cycle progression. Niclosamide markedly increased cell population at G1 phase (60% in control *vs*. 75% in niclosamide treated cells), but decreased the percentage of cells at S and G2/M phases (control *vs*. niclosamide-treated cells, S: 23.90 ± 0.17 *vs*. 10.27 ± 0.26; G2/M: 15.04 ± 0.12 *vs*. 12.64 ± 0.09, mean ± SEM (*n* = 3), *p* < .001) in cPARP-negative live cell population, indicating that niclosamide inhibits AML cell viability by inducing cell cycle arrest at G1 phase as well as apoptosis (Figure 5A).

To study the effects of niclosamide on CREB-target genes, we examined the expression of *RFC3* and *POLD2*, which are factors involved in DNA replication and repair [35]. CREB regulates G1/S cell cycle transition by controlling *RFC3* expression [36]. In addition, mRNA levels of *RFC3* and *POLD2* are downregulated by CREB knockdown and treatment with CREB inhibitors in AML cells [17, 36, 37]. Quantitative RT-PCR data showed that niclosamide significantly decreased mRNA expression levels of *RFC3* and *POLD2* in HL60 cells (Figure 5B), suggesting that niclosamide downregulates CREB-target genes to induce apoptosis and cell cycle arrest in AML cells.

### *In vivo* efficacy of niclosamide in AML-PDX mice

**Figure 6:**
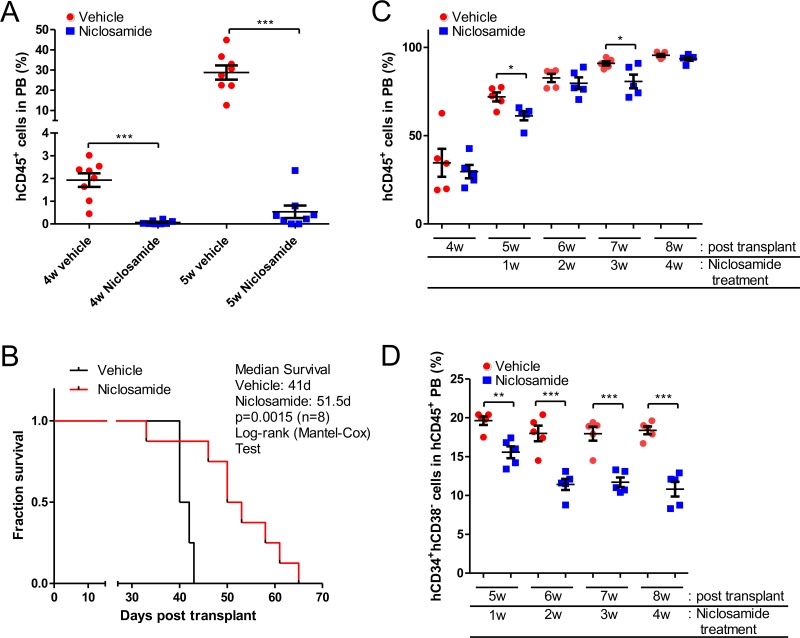
*In vivo* efficacy of Niclosamide in PDX mice **A.**, **B.** Prolonged survival of AML-PDX (186) mice. Sub-lethally irradiated NSG mice were injected with 1 × 10^6^ patient AML (186) cells. Niclosamide (200mg/kg) was given orally daily starting the day after cell injection, until death or when an endpoint was reached. Leukemia development was monitored by quantifying the fraction of human CD45-positive (hCD45^+^) cells in peripheral blood (PB) of AML-PDX (186) mice using flow cytometry. Niclosamide treatment results in decreased numbers of circulating AML cells (mean ± SEM, *n* = 8, *p* > 0.001) (A). Survival of AML-PDX (186) mice treated with niclosamide was monitored over time, and is presented by a Kaplan-Meier plot. Median survival for niclosamide was 51.5 days compared to vehicle control of 41 days (*n* = 8, *p* = 0.0015, log-rank test) (B). **C.**, **D.** Suppression of hCD34^+^hCD38^-^ leukemia stem cell (LSC) population in AML-PDX (3123) mice by niclosamide treatment. NSG mice were injected with 1 × 10^6^ patient AML cells (3123). Niclosamide (200mg/kg) were given once daily by oral gavage starting 4 weeks after cell injection, until death or an endpoint was reached. (C) To monitor the leukemia disease progression, PB was analyzed to quantify the fraction of human hCD45^+^ cells in AML-PDX (3123) mice. Each spot represents an individual mouse. Data are graphed as mean ± SEM (*n* = 5). (D) hCD34^+^hCD38^-^ LSC population of circulating human AML cells was determined by flow cytometry. Data are graphed as mean ± SEM (*n* = 5). *, *p* < .05; **, *p* < .001; ***, *p* < .0001.

To study the efficacy of niclosamide in AML patient-derived xenograft (PDX) mouse models, we generated PDX models by injecting AML cells from patients into immune-deficient mice [38]. NOD.Cg-*Prkdc^scid^* *Il2rg^tm1Wjl^*/SzJ (NSG) mice were transplanted with one million primary AML (186) cells. Mice were treated with niclosamide or vehicle control once daily by oral gavage starting the day after cells were injected. Leukemia disease progression was monitored by measuring the fraction of human CD45^+^ cells in the peripheral blood (PB) of AML-PDX (186) mice using flow cytometry. Niclosamide significantly inhibited the progression of AML in AML-PDX (186) mice. While vehicle-treated mice showed a rapid increase of circulating leukemia cells in PB, niclosamide treatment suppressed the percentage of circulating AML cells to less than 1% until 5 weeks after transplantation [hCD45^+^ cells in PB (%), vehicle *vs*. niclosamide treatment 5 weeks after engraftment, 28.75 ± 3.51 *vs*. 0.54 ± 0.27 (*n* = 8, *p* < 0.001, mean ± SEM)] (Figure 6A). Consistent with this inhibitory effect on development of leukemia, niclosamide significantly improved the survival of mice. In Kaplan Meier analysis, the median survival of PDX mice was 41 days *vs*. 51.5 days (*p* = 0.0015, log-rank test) (Figure 6B).

We evaluated the *in vivo* efficacy of niclosamide with a second AML patient sample (3123). Since AML-PDX (3123) showed a rapid disease onset and a longer median survival, we started treatment 4 weeks after transplantation of leukemia when most mice had an increased leukemic burden of over 20% for a more clinically relevant experimental model. In this model, niclosamide significantly reduced the leukemic burden more than vehicle control after 1w of treatment temporarily [hCD45^+^ cells in PB (%), vehicle *vs*. niclosamide, 71.92 ± 2.56 *vs*. 61.18 ± 2.50 (*n* = 5, *p* < 0.05, mean ± SEM)] (Figure 6C). The median survival of mice was improved compared to vehicle (vehicle: 67 days *vs*. niclosamide: 72 days), but could not reach statistical significance. Surprisingly, CD34^+^CD38^-^ leukemic stem cell (LSC) population significantly declined following treatment of niclosamide [CD34^+^CD38^-^ LSC in circulating leukemic cells (%), vehicle *vs*. niclosamide, after 1 week of treatment, 19.62 ± 0.54 *vs*. 15.56 ± 54.48; 4 weeks of treatment: 18.38 ± 0.50 *vs*. 10.81 ± 0.95 (*n* = 5, *p* < 0.01, mean ± SEM)] (Figure 6D).

### Enhanced chemotherapy sensitivity by niclosamide pretreatment

**Figure 7:**
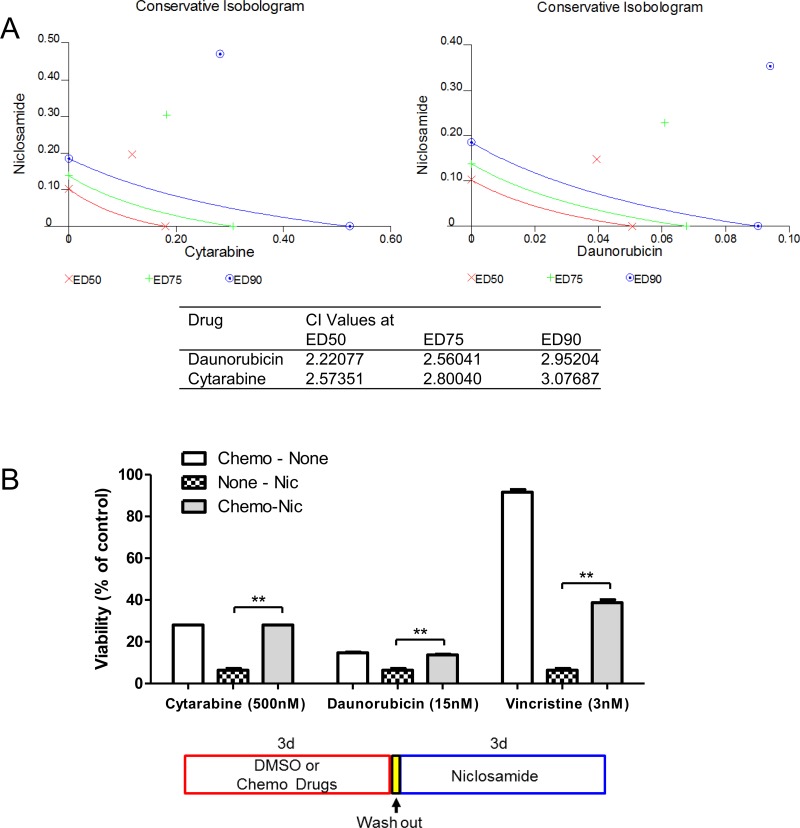
Inhibitory effect of chemotherapy drugs on cytotoxic response to niclosamide **A.** Antagonistic effect of simultaneous treatment with niclosamide and chemotherapy drugs cytarabine and daunorubicin. Cells were treatment with different doses of niclosamide and chemotherapy drugs for 3d. Conservative isobolograms and CI values were generated by Chou-Talalay method using CalcuSyn software. **B.** Pretreatment of chemotherapy drugs resulted in resistance to niclosamide. HL60 cells were treated with cytarabine, daunorubicin, or vincristine for 3d, washed with culture medium twice, and then treated with niclosamide for 3d. Cell viability was measured using CellTiter-Glo assay. The combination of chemotherapy drugs and niclosamide was compared with niclosamide alone. A diagram shows the experimental scheme. Data are graphed as mean ± SEM (*n* = 3). **, *p* < .001

**Figure 8:**
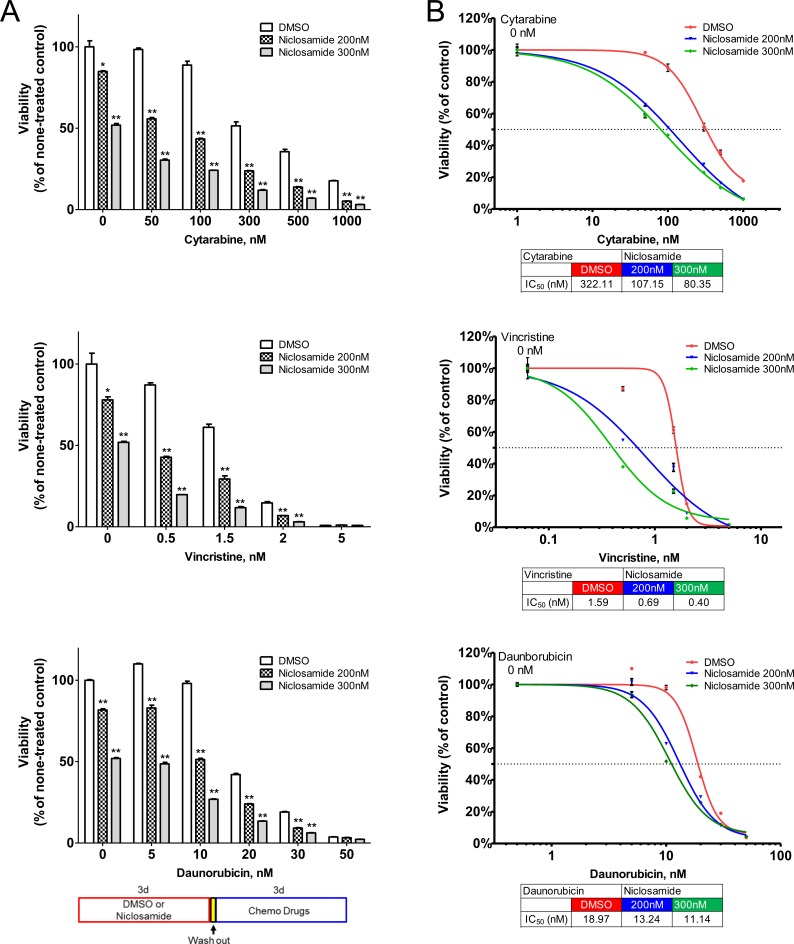
Synergistic cytotoxic effect of sequential treatment with niclosamide and chemotherapy drugs cytarabine, daunorubicin, and vincristine HL60 cells were treated with niclosamide (200nM, 300nM) or DMSO for 3 days. After cells were washed with culture medium twice, cells were further incubated with various doses of chemotherapy drugs or vehicle for 3 days. **A.** Pretreatment of cells with niclosamide significantly potentiated the cytotoxic effects of chemotherapy drugs. Cell viability was measured using CellTiter-Glo assay and expressed as the percentage of none-treated control cells. The sequential combination of niclosamide and chemotherapy drugs was compared to the effect of chemotherapy drugs alone. The diagram shows the experimental scheme. Results are represented as mean ± SEM (*n* = 3). *, *p* < .05; **, *p* < .001. **B.** Pretreatment with niclosamide sensitized cells to chemotherapy. The graphs show representative dose response curves. Dose response curves of chemotherapy drugs are shifted to the left with pretreatment of niclosamide. The IC_50_ values are indicated below the graphs. Values are presented as mean ± SEM (*n* = 3).

The classical frontline treatment of AML is the “7+3” combination induction chemotherapy which consists of 7 days of cytarabine and 3 days of an anthracycline, most often daunorubicin [1]. We investigated the efficacy of niclosamide combined with chemotherapy. To assess the simultaneous treatment effects of niclosamide and chemotherapy drugs (cytarabine, daunorubicin), HL60 cells were treated with combined drugs at a fixed ratio of IC_50_ for niclosamide and chemotherapy drugs. The simultaneous treatment of HL60 cells with niclosamide and cytarabine or daunorubicin had antagonistic effects on cellular viability of HL60 cells with CI values of >2 (Figure 7A). Isobolograms for both combinations showed combination values to be located far upper right of the additive isoboles. We next tested a sequence-dependent combination with niclosamide and chemotherapy. Though vincristine is not used in current AML therapy, it was included in combination chemotherapy of the VAPA study, one of the first AML studies in children and adults [39]. We examined whether chemotherapy inhibited cytotoxic potency of niclosamide. HL60 cells were treated with chemotherapy drugs (cytarabine, daunorubicin, or vincristine) and niclosamide sequentially. Several other chemotherapy drugs including decitabine, 5-azacytidine, mitoxantrone were tested for potential efficacy in combination with niclosamide (data not shown). Pretreatment of cells with chemotherapy (cytarabine, daunorubicin, or vincristine) reduced cytotoxic effects of niclosamide (Figure 7B). We then investigated whether niclosamide could potentiate the activity of chemotherapy. HL60 cells were pretreated with niclosamide for 3 days prior to treatment with various doses of chemotherapeutic drugs (cytarabine, daunorubicin, or vincristine) for another 3 days. Pretreatment of cells with niclosamide followed by chemotherapeutic drugs showed a synergistic effect. The sequential combination treatment of niclosamide and chemotherapy drugs significantly lowered the viability of cells compared to chemotherapy drugs alone (Figure 8A). Furthermore, pretreatment with niclosamide caused dose-curves of chemotherapeutic drugs to shift to the left with lower IC_50s_, suggesting that pretreatment with niclosamide conferred sensitivity to these chemotherapeutic agents even at suboptimal doses (Figure 8B).

## DISCUSSION

We previously demonstrated that CREB overexpression increases leukemia cell growth and survival [6, 13], and inhibition of CREB by a small molecule, XX-650-23, or shRNA induces cell cycle arrest and apoptosis in AML cells [14, 17, 36]. The tool compound XX-650-23 inhibits CREB transcriptional activity, leading to suppression of viability of AML cells *in vitro* and prolonged survival of AML xenograft mice with no toxicity [17]. However, the potency, solubility, and half-life of XX-650-23 are not adequate for clinical application. In this study, we demonstrated that niclosamide, a well-tolerated FDA-approved oral anthelminthic drug, selectively blocked CREB activation, disrupted CREB-CBP interaction, and inhibited CREB-driven genes expression, resulting in inhibition of AML cell viability *in vitro* and leukemia progression in AML-PDX mice.

In recent studies, niclosamide has been identified as a potent anticancer drug in a broad range of tumor cells [20, 23–25]. In addition, niclosamide inhibits cell proliferation of NCI-60 human tumor cell lines with IC_50_ of less than 1 μM [23]. Though niclosamide has been reported to target several signaling pathways including Wnt/β-catenin, mTORC1, STAT3, NF-κB and Notch [20, 23–25, 34, 40], the mechanism of action of niclosamide in AML has not been extensively studied.

A previous report described inhibition of tumor necrosis factor (TNF)–induced NF-κB activation as a potential mechanism of niclosamide action in AML cells [25]. However, without TNF stimulation, NF-κB activation was not observed in our results and niclosamide did not show any further inhibition of NF-κB. Phosphorylation at Ser536 of NF-κB p65 requires IκB kinase (IKK), p38, or ribosomal S6 kinase 1 (RSK1) [41], but niclosamide has not been reported to inhibit 95 kinases including NF-κB p65 kinase up to 10 μM in *in vitro* kinase assays [40]. Recently, Tang *et al*. showed that niclosamide derivatives did not show the correlation between anti-proliferative effects with inhibition of NF-κB or mitochondrial transmembrane potential in HL60 cells [42]. We did not observe the reduction of p- NF-κB p65 (S536)-positive population in HL60 and KG1 cells following treatment of niclosamide (Figure 4B), consistent with the previous *in vitro* IKK assay [40]. STAT3 was inhibited only in KG1 cells, but not in HL60 cells, following treatment of niclosamide. Based on our data and recent reports, CREB appears to be a potential target of niclosamide in AML cells.

While the oral bioavailability of niclosamide was only 10% in rats due to its poor solubility and absorption [43], niclosamide is well-tolerated in mammals (1000 or 2500 mg/kg b.w. oral daily for 55 or 64 days, thereafter 10,000 and 25,000 mg/kg feed; total duration 365–381 days in male rats without any damage based on clinical symptoms, hematolology/clinical chemistry tests, and histopathological examination) [18]. Our data first demonstrated that oral administration of niclosamide significantly inhibited AML progression in PDX mice (Figure 6). A single 2 g oral dose of niclosamide to adults for cestocidal treatment or a single 5 mg/kg oral dose to rats leads to maximum plasma concentrations of 0.76–18.35 μM [18] and 1.08 μM [43], respectively. These plasma concentrations should be sufficient to suppress AML cell viability in humans or increase sensitivity of AML cells to chemotherapy. Niclosamide is cleared rapidly from plasma. Plasma concentration decrease to 0.11 μM, below active dose, 2 hours after oral administration in rats [43]. Since there is a high tolerability of repeated high doses of niclosamide, clinical active plasma concentration of niclosamide can be maintained safely.

Schedule-dependent synergy was observed when we tested the effects of niclosamide and chemotherapy drugs, cytarabine, daunorubicin, or vincristine. Chemotherapy-induced DNA damage activates the cell cycle checkpoints to stop cell cycle progression for DNA repair by activating DNA damage response (DDR) signaling pathways, ATM/ATR–CHK1/CHK2–p53 axis [44]. We postulate that activate DDR pathways turn on the DNA repair and survival proteins, which could increase the threshold to trigger cell death by niclosamide treatment. Niclosamide reduced expression of *RFC3* and *POLD2*, which support this hypothesis. RFC3 is required for DNA replication, DNA repair, and DDR checkpoint control [45]. RFC complexes function to load clamp proteins, proliferating cell nuclear antigen (PCNA) or checkpoint 9-1-1 complex (Rad9-Rad1-Hus1) on chromatin [46]. PCNA provides a platform for DNA metabolism-related proteins to process DNA replication, DNA repair, and cell cycle control [47–50]. Rad17-RFC loads the 9-1-1 complex to activate ATR, which turns on DDR signaling and cell cycle checkpoints [46, 51]. CREB regulates RFC3 expression to control G1/S progression by modulating chromatin loading of PCNA in AML cells [36]. DNA polymerase delta is also an essential component for DNA replication and DNA repair [52]. Downregulation of these CREB-driven genes could diminish the capacity of DDR and DNA repair, which lowers threshold to turn on cell death pathway following chemotherapy. Taken together, our study provides preclinical evidence that supports a possible strategy of incorporating niclosamide as a single agent or in combination with chemotherapy to treat AML patients. Furthermore, lowering the dose of chemotherapy used in combination with niclosamide could prevent some of the known toxicities and long-term complications observed with AML therapy. This is particularly relevant for AML patients in third world countries where supportive care is severely limited. Therefore, our study provides a potentially novel approach to treat AML patients and needs further investigation in clinical trials.

## MATERIALS AND METHODS

### Cell culture

Human acute myeloid leukemia cells were cultured at 37°C with 5% CO_2_ in following medium; IMDM/10% fetal bovine serum (FBS)/1% penicillin/streptomycin/glutamine (PSG) for HL60, KG1, and MV4-11 cells, RPMI1640/20% FBS/1% PSG for MOLM13 cells, RPMI1640/10%FBS/1% PSG for U937 cells. Cell lines were obtained from ATCC (Manassus, VA, USA) and low-passage stocks were used and cultured for less than 3 months maintained. Cells were regularly tested for Mycoplasma and growth characteristics. Primary AML cells were cultured in IMDM/15% BIT (bovine serum albumin, insulin, transferrin; Stem Cell Technologies, Vancouver, BC, Canada)/SCF (100 ng/ml, Miltenyi Biotec, Auburn, CA, USA)/FLT3-ligand (50 ng/ml, Miltenyi Biotec)/IL3 (20 ng/ml, Miltenyi Biotec)/IL6 (20 ng/ml, Miltenyi Biotec)/TPO (50 ng/ml, Miltenyi Biotec)/GMCSF (20 ng/ml, R&D Systems, Minneapolis, MN, USA)/55 μM β-mercaptoethanol/0.75 μM SR1 (Selleck Chemicals, Houston, TX, USA)/35 nM UM171 (Stem Cell Technologies), as described previously [53, 54]. Bone marrow from AML patients were collected through voluntary patient participation at Stanford University (Palo Alto, California, USA) in compliance with the Institutional Review Board regulations. Informed consent was obtained from all human subjects in accordance with the declaration of Helsinki and the Data Protection Directive. HL60 cells were transduced with lentivirus expressing CREB shRNAs [14] to generate CREB knockdown cells. Lentivirus-infected cells were isolated using a FACS Aria (BD Biosciences, San Jose, CA, USA) 3d after transduction.

### Cell viability and apoptosis analysis

For cell viability assays, AML cell lines and primary AML cells were seeded at 2x 10^4^ cells/well in 96-well plates, and then treated with niclosamide for 3 or 4 days, respectively. CellTiter-Glo assays (Promega, Madison, WI, USA) were performed to assess viability of cells. The software program GraphPad Prism version 5.0 (GraphPad Software, La Jolla, CA) was used to determine the concentration inhibiting cell viability by 50% (IC50). For combination experiments, combination index values were calculated by Chou-Talalay method using CalcuSyn software (Biosoft, Ferguson, MO, USA) as described [55]. CI values were used to determine synergism (CI<1), antagonism (CI>1) and additivity (CI=1). Cells were treated with combined drugs at a constant ratio of IC50 for two drugs. Additivity ranges of CI scores were also determined from sham mixtures.

Apoptosis was measured by Annexin-V (Biolegend, San Diego, CA, USA) / 4’,6-diamidino-2-phenylindole (DAPI) double staining according to manufacturer's instructions. Stained cells were analyzed on a DxP10 FACScan (BD Biosciences/Cytek Development, Fremont, CA, USA).

### Hematopoietic cell colony assays

Normal human bone marrow cells or primary AML cells were plated in methylcellulose (2×10^4^ cells/mL, Miltenyi Biotec) containing cytokines (GM-CSF, G-CSF, IL-3, IL-6, SCF, erythropoietin) to assess colony-forming cell units. Colonies were scored on the basis of morphology after 2 weeks of culture.

### RNA extraction and quantitative reverse transcription PCR (qRT- PCR)

mRNA expression was assessed by quantitative reverse transcription PCR as described previously [17, 36]. Total RNA was extracted from cells using QIAGEN RNAeasy Mini Kit (Qiagen, Valencia, CA, USA) according to the manufacturer's instructions and used for cDNA synthesis by iScript cDNA Synthesis Kit (BioRad, Hercules, CA, USA). PCR was carried out on a CFX384 Real-time PCR system (BioRad) using IQ™ SYBR® Green Supermix (BioRad). The β-actin was used as a control gene. Relative expression levels were calculated as 2^−ΔΔCT^ [56].

### CREB-dependent transcription and KIX-KID interaction assays

To determine the inhibitory effect of niclosamide on CREB-dependent transcription, HL60 cells expressing a Firefly luciferase under CREB-driven promoter was generated, as previously described [17, 37]. Cells were seeded at 7.5x 10^4^ cells/well in 96-well plates, and then cultured with various concentrations of niclosamide for 6h. Bright Glo assay system (Promega) was used to measure Firefly luciferase activity. KIX-KID interaction was monitored using the split Renilla luciferase complementation assay [57]. HEK 293 cells were transfected with vectors expressing KID domain fused to N-terminal Renilla luciferase and KIX domains fused to C- terminal Renilla luciferase using TransIT-293 transfection reagent (Mirus Bio, Madison, WI, USA). Next day, transfected cells were replated in 96-well plates. Cells were allowed to attach overnight, then treated with different doses of compounds 0.5h before stimulation with forskoin (6 μM). Cells were further cultured for 1.5h. Subsequently, culture media was discarded, and then 100 μl of phosphate-buffered saline (PBS) containing coeletarazine (20 μM) was added to determine Renilla luciferase activity.

### Flow cytometry analysis

For detection of intracellular proteins by flow cytometry, cells were fixed with 1.6% paraformaldehyde for 10 min, and then post-fixed in cold-methanol at -70^°^C. Fixed cells were washed and incubated with antibodies and DAPI (0.1ug/ml, Sigma, St. Louis, MO, USA) in PBS with 1% BSA. The following antibodies were used in intracellular protein staining: PE-conjugated anti-Cyclin A (clone BF683), Alexa Fluor 647-conjugated anti-cPARP (clone F21-852), PE-conjugated anti-pCERB (pS133) (clone J151-21), Alexa Fluor 488-conjugated anti-pSTAT1 (pY701) (clone 4a), PE-conjugated anti-pSTAT3 (pY705) (clone 4/P-STAT3), Alexa Fluor 647-conjugated anti-pSTAT5 (pY694) (clone 47, BD Biosciences); PECy7-conjugated ant-pH3 (pS28) (clone HTA283, Biolegend); Alex Fluor 647-conjugated anti-p-NFkB p65 (pS536) (clone 93H1, Cell Signaling Technology, Danvers, MA, USA).

To assess engrafted cell populations with cell surface markers, cells were stained with FITC-conjugated anti-hCD34 (AC136, Miltenyi Biotec), PE-conjugated anti-hCD38 (HB7, BD Biosciences), and APC-conjugated anti-hCD45 (HI30, Biolegend) antibodies. DAPI was included in staining solutions to remove dead cell population. Cells were analyzed on FACSCalibur (BD Biosciences) or DxP10 FACScan (BD Biosciences/Cytek Development).

### AML PDX mice

All mouse experiments were subject to institutional approval by Stanford University Institutional Animal Care and Use Committee. For AML PDX mice experiments, patient primary AML cells (1 × 10^6^ per mouse) were injected in to sub-lethally irradiated NOD.Cg-*Prkdc^scid^* *Il2rg^tm1Wjl^*/SzJ (NSG) mice (primary xenograft mice) for engraftment. Patient AML cells from primary xenograft mice were injected into secondary xenograft mice to expand patient AML cells. Cells from secondary xenograft mice were injected into sub-lethally irradiated NSG mice for *in vivo* efficacy test of niclosamide. Engrafted mice were randomly divided into two groups. Mice were separated into two groups of 200mg/kg niclosamide (formulated in 0.5% w/v methylcellulose/0.1% v/v Tween 80) treatment and vehicle. The treatment was given once daily by oral gavage starting 24 hours after cell infection or 4 weeks after cell injection. Leukemia disease progression in mice was monitored by flow cytometry analysis of circulating leukemic cells in PB. After lysing red blood cells, cells were stained with anti-hCD45, hCD34, hCD38 antibodies. Percentage of each population was measured on a flow cytometer. The overall survival of mice was monitored to determine the median survival.

### Statistical analysis

Unless otherwise stated, all experiments were performed in triplicate, and statistical significance was determined using the unpaired two-tailed Student's t-test. Kaplan-Meier plots and statistical significance of differences in mice survival experiments were calculated using a Log-rank (Mantel-Cox) test with Prism. Isobologram analysis for combination treatment analysis was performed using the CalcuSyn software program.
